# Cerebral Sinovenous Thrombosis Mimicking Intracranial Mass

**DOI:** 10.4274/tjh.2016.0038

**Published:** 2016-08-19

**Authors:** Derya Özyörük, Neslihan Karakurt, Arzu Yazal Erdem, Suna Emir, Bahattin Tunç, Neşe Yaralı, Namık Özbek

**Affiliations:** 1 Ankara Children’s Hematology and Oncology Training and Research Hospital, Ankara, Turkey

**Keywords:** Intracranial mass, Cerebral sinovenous thrombosis, increased intracranial pressure

## To the Editor,

Cerebral sinovenous thrombosis is rare in children [1]. Most common signs and symptoms are seizure, lethargy, and headache [1,2,3]. We herein report a case diagnosed as cerebral sinovenous thrombosis mimicking an intracranial mass and presenting with increased intracranial pressure symptoms in an adolescent girl.

**Figure 1:**
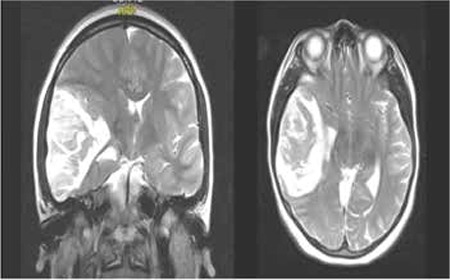
Cranial magnetic resonance images of the patient demonstrate a heterogeneous parenchymal lesion with increased T2 signal intensity in the right temporal lobe causing compression of the third lateral ventricle, basal ganglion, and thalamus with sylvian fissure, sulcal effacement, shifting midline structure, and significant edema.

A 16-year-old girl was admitted to our emergency service with complaints of headache, vomiting, and confusion. Her past medical history was unremarkable. Physical examination revealed facial paralysis and motor weakness on the left side. Magnetic resonance (MR) imaging disclosed a mass (65x42x55 mm) in the right temporal lobe shifting the midline structure from right to left (Figure 1). Because of herniation findings, surgery was performed immediately. Histopathologic investigation showed hematoma, gliosis, inflammation, and vasculitis. The laboratory examination revealed iron deficiency anemia (white blood cells: 12x109/L, Hb: 7.2 g/dL, mean corpuscular volume: 49 fL, red cell distribution width: 20%, platelets: 570x109/L, ferritin: 4.4 ng/mL). Other hematologic tests and coagulation panels were determined to be normal. Prothrombotic markers such as protein C, protein S, antithrombin III, plasminogen, heparin cofactor II, factor VIII, factor XII, lipoprotein (a), fibrinogen, homocysteine, anticardiolipin IgG, lupus anticoagulant levels, prothrombin 20210G, and factor V Leiden were normal. Tumor markers were negative. Cranial MR venography demonstrated stasis and occlusion in the middle distal part of the transverse sinus, sigmoid sinus, and internal jugular vein. The patient had no signs of nephrotic syndrome, infection, or vasculitis. She was diagnosed with transverse sinus thrombosis and anticoagulated with low-molecular-weight heparin. Iron supplementation was administered for six months.

Cases of thrombosis presenting with signs of intracranial mass are rarely reported in the literature. Kim et al. [4] reported a 54-year-old male patient who was operated on for an intracranial tumor; however, the final diagnosis was thrombus in the aneurysm of the middle cerebral artery. In another report, a 20-month-old girl presented with rapidly progressing hemiparesis on the left side. Cranial MR revealed an abscess or glial tumor-like lesion in the right thalamus; however, pathological investigation of the stereotactic biopsy specimen did not show any signs of malignancy. On day 14 of admission, control cranial MR showed thrombosis in the superior sagittal sinus and transverse sinus [5].

The association of iron deficiency anemia with sinus thrombosis has been reported previously in children [6]. Although secondary thrombocytosis has been implicated in cerebral venous sinus thrombosis associated with iron deficiency, two cases with normal platelet count have also been described [7]. As not all cases of iron-related thrombotic events occur in patients with concomitant high platelet counts, other pathogenic mechanisms have been proposed. One of these explanations is that iron deficiency may contribute to a hypercoagulable state by affecting blood flow patterns within the vessels because of reduced deformability and increased viscosity of microcytic red blood cells. Furthermore, anemic hypoxia secondary to iron deficiency has been suggested to precipitate situations of increased metabolic stress, in particularly in vulnerable areas of the brain supplied by end arteries [8]. In our patient, iron deficiency may also have contributed to the development of thrombosis.

In conclusion, cerebral sinovenous thrombosis associated with increased intracranial pressure symptoms may mimic intracranial masses in children.
